# Inositol hexa*kis*phosphate biosynthesis underpins PAMP‐triggered immunity to *Pseudomonas syringae* pv. *tomato* in *Arabidopsis thaliana* but is dispensable for establishment of systemic acquired resistance

**DOI:** 10.1111/mpp.12902

**Published:** 2019-12-26

**Authors:** Jacquelyne S. Y. Poon, Ruth E. Le Fevre, John P. Carr, David E. Hanke, Alex M. Murphy

**Affiliations:** ^1^ Department of Plant Sciences University of Cambridge Cambridge United Kingdom

**Keywords:** *Arabidopsis thaliana*, basal resistance, biosynthesis, inositol hexa*kis*phosphate, Ins*P*_6_, PAMP‐triggered immunity, *Pseudomonas syringae* pv. *tomato*, systemic acquired resistance

## Abstract

Phytic acid (inositol hexa*kis*phosphate, Ins*P*_6_) is an important phosphate store and signal molecule necessary for maintenance of basal resistance to plant pathogens. *Arabidopsis thaliana* (‘arabidopsis’) has three genes encoding *myo*‐inositol phosphate synthases (IPS1–3), the enzymes that catalyse conversion of glucose‐6‐phosphate to Ins*P*, the first step in Ins*P*_6_ biosynthesis. There is one gene for inositol‐(1,3,4,5,6)‐penta*kis*phosphate 2‐kinase (IPK1), which catalyses the final step. Previously, we showed that mutation of *IPS2* and *IPK1* but not *IPS1* increased susceptibility to pathogens. Our aim was to better understand the Ins*P*_6_ biosynthesis pathway in plant defence. Here we found that the susceptibility of arabidopsis (Col‐0) to virulent and avirulent *Pseudomonas syringae* pv. *tomato* was also increased in *ips3* and *ips2/3* double mutants. Also, *ipk1* plants had compromised expression of local acquired resistance induced by treatment with the pathogen‐derived molecular pattern (PAMP) molecule flg22, but were unaffected in other responses to flg22, including Ca^2+^ influx and the oxidative burst, seedling root growth inhibition, and transcriptional up‐regulation of the PAMP‐triggered genes *MITOGEN‐ACTIVATED PROTEIN KINASE* (*MPK*) *3*, *MPK11*, *CINNAMYL ALCOHOL DEHYDROGENASE 5*, and *FLG22‐INDUCED RECEPTOR‐LIKE KINASE 1*. *IPK1* mutation did not prevent the induction of systemic acquired resistance by avirulent *P. syringae*. Also, *ips2* and *ips2/3* double mutant plants, like *ipk1*, were hypersusceptible to *P. syringae* but were not compromised in flg22‐induced local acquired resistance. The results support the role of Ins*P*_6_ biosynthesis enzymes in effective basal resistance and indicate that there is more than one basal resistance mechanism dependent upon Ins*P*_6_ biosynthesis.

## Graphical abstract

Mutating *Inositol‐Pentakisphosphate 2‐Kinase* (*IPK1*) compromises PAMP‐triggered immunity. Activity of inositol phosphate synthases IPS2 and IPS3 supports other basal resistance phenomena.

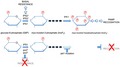

## 1 INTRODUCTION

Phytic acid (*myo*‐inositol‐1,2,3,4,5,6‐hexa*kis*phosphate: Ins*P*_6_) is a phosphorylated derivative of *myo*‐inositol that is ubiquitous in eukaryotes. In plants, Ins*P*_6_ is found abundantly in seeds and is thought to be an important reserve for phosphorus, *myo*‐inositol, and minerals (Lott *et al*., 2000; Otegui *et al*., 2002). Ins*P*_6_ is also involved in the mediation of drought stress responses triggered by the hormone abscisic acid by controlling release of Ca^2+^ from endomembrane stores in guard cells (Lemtiri‐Chlieh *et al*., 2003). Subsequently, it was shown that the Ins*P*_6_ biosynthesis pathway is required for defence against pathogens (Murphy *et al*., 2008). Ins*P*_6_ is a cofactor for the auxin receptor TIR1 (transport inhibitor response 1) in conjunction with the SCF^TIR1^ adaptor ASK1 (Tan *et al*., 2007), whilst other inositol polyphosphate species, Ins(1,2,4,5,6)*P*_5_ and Ins*P*_8_, have been proposed to be cofactors for the jasmonic acid (JA) co‐receptor COI1 (Sheard *et al*., 2010; Laha *et al*., 2015). These findings indicate that Ins*P*_6_ and related inositol polyphosphates have important biological functions in plants.

Ins*P*_6_ biosynthesis can occur in either a lipid‐dependent or a lipid‐independent manner (Figure S1). In the lipid‐dependent pathway, phosphatidylinositol species are synthesized, and hydrolysis of phosphatidylinositol‐(4,5)‐bisphosphate by phospholipase C releases Ins(1,4,5)*P*_3_ (Brearley *et al*., 1997), which is subsequently phosphorylated by inositol polyphosphate kinases (Stevenson‐Paulik *et al*., 2002). Within the lipid‐independent pathway described in plants, the enzyme *myo*‐inositol phosphate synthase (IPS) catalyses the conversion of glucose‐6‐phosphate to Ins*P* (Loewus *et al*., 1980, 1982). Ins*P* is sequentially phosphorylated by inositol polyphosphate kinases, leading to the final phosphorylation of the 2‐OH of the *myo*‐inositide, catalysed by inositol‐(1,3,4,5,6)‐penta*kis*phosphate 2‐kinase (IPK1) to generate Ins*P*_6_ (González *et al*., 2010; Baños Sanz *et al*., 2012).

Ins*P*_6_ has a strong affinity for divalent metal ions due to its six phosphate groups. This property gives dietary Ins*P*_6_ antinutrient properties in monogastric animals, including humans, because it chelates minerals and prevents their uptake by the gut. This is especially deleterious for people with mineral‐deficient diets (Cheryan and Rackis, 1980). Ins*P*_6_ can also cause environmental damage. This arises when Ins*P*_6_ in feed passes undigested through monogastric livestock (pigs, for example) and enters nearby watercourses, in which it can promote excessive algal growth, leading to eutrophication (Turner *et al*., 2002). The negative health and environmental impacts associated with Ins*P*_6_ have provided an incentive to breed low phytic acid crops (Raboy, 2002). Hence, a variety of low phytic acid mutant lines have been investigated in maize, barley, and rice (Raboy *et al*., 2001).

However, given the important biological functions of Ins*P*_6_ in plants, deployment of crops with total depletion of Ins*P*_6_ should be done cautiously. Plants disrupted in the Ins*P*_6_ biosynthesis pathway exhibit defects in plant development, abiotic stress tolerance, and biotic stress responses. *Arabidopsis thaliana* (hereafter ‘arabidopsis’) mutant plants with a T‐DNA insertion within the *IPK1* gene (locus At5g42810) appear stunted under long day conditions, exhibit an early flowering phenotype (Lee *et al*., 2015), and have low levels of Ins*P*_6_ in seeds (Stevenson‐Paulik *et al*., 2002; Kim and Tai, 2011) and vegetative tissue (Kuo *et al*., 2018). In arabidopsis, IPS enzymes are encoded by a small family of three genes: *IPS1* (At4g39800), *IPS2* (At2g22240), and *IPS3* (At5g10170). Arabidopsis *ips1* mutants are depleted in *myo*‐inositol (Donahue *et al*., 2010) but not in Ins*P*_6_ (Kuo *et al*., 2018). The *ips1* mutants, but not *ips2* or *ips3* mutants, have defects in plant development, characterized by curling and spontaneous lesions in leaves and distorted root caps (Meng *et al*., 2009; Chen and Xiong, 2010; Donahue *et al*., 2010). Arabidopsis *ips1* mutants also exhibit impaired auxin transport in roots in a PIN2‐dependent manner (Chen and Xiong, 2010). Furthermore, arabidopsis *ips2*, but not *ips1*, mutant plants are hypersusceptible to a range of pathogens, including several RNA viruses and one DNA virus, the hemibiotrophic bacterial pathogen *Pseudomonas syringae* pv. *tomato* (Pst), and the necrotrophic fungus *Botrytis cinerea* (Murphy *et al*., 2008). Likewise, *ipk1* mutant arabidopsis plants are also compromised in basal resistance to viruses, bacteria, and the cyst nematode *Heterodera schachtii* (Murphy *et al*., 2008; Jain *et al*., 2015; Ma *et al*., 2017). The weakened resistance seen in *ips2* and *ipk1* mutant plants supported a role for the Ins*P*_6_ biosynthetic pathway in pathogen defence, yet it was unknown whether *IPS3* is also involved in biotic stress responses.

Plants have evolved layers of defence to combat microbial pathogens. The initial layer is characterized by the perception and recognition of pathogen‐associated molecular patterns (PAMPs) by surface‐localized pattern recognition receptors (PRRs), leading to PAMP‐triggered immunity (PTI) (Boller and Felix, 2009). A classical PAMP recognized by a PRR is a sequence of 22 conserved amino acid residues in bacterial flagellin (flg22), which is perceived by the LRR‐RLK receptor FLS2 (flagellin sensing 2) (Boller and Felix, 2009). FLS2‐mediated perception of flg22 and its downstream signalling effects have become a paradigm for PTI in plants (reviewed in Boller and Felix, 2009). For instance, PAMP treatment triggers the rapid release of reactive oxygen species (ROS) (Felix *et al*., 1999; Kunze *et al*., 2004), a characteristic influx of cytosolic Ca^2+^ (Ma and Berkowitz, 2011), a cascade of sequential MAPK phosphorylation (Jagodzik *et al*., 2018), and a series of transcriptional changes (Clay *et al*., 2009). In turn, virulent pathogens have evolved specific avirulence (Avr) effectors to suppress a variety of PTI‐related processes. In the case of Pst these are secreted into the host cell through the Type III secretion injectisome (Arnold and Jackson, 2011; Block and Alfano, 2011).

Resistant host plants can recognize Avr effectors if they possess appropriate cytoplasmic NB‐LRR resistance (R) proteins. Recognition results in an accelerated, amplified resistance response termed effector‐triggered immunity (ETI) or the hypersensitive response (Carr *et al*., 2010). Elicitation of ETI can activate throughout the plant an enhanced state of readiness to inhibit infection upon any subsequent pathogen attack, a state called ‘systemic acquired resistance’ (SAR) (Carr *et al*., 2010). Effector recognition can occur indirectly, as is the case when NB‐LRR proteins detect effector‐induced changes in the host cell (Carr *et al*., 2010). For instance, in arabidopsis the Pst AvrRPT2 effector cleaves RIN4 (RPM1‐interacting protein), which can be detected by the R proteins, RPS2 and RPM1 (Kim *et al*., 2009). Another Pst effector, AvrB, phosphorylates the host protein RIN4 (Mackey *et al*., 2002). This leads to enhanced host susceptibility by up‐regulating JA‐dependent responses (He *et al*., 2004) in a manner dependent upon the JA co‐receptor, COI1 (Shang *et al*., 2006), and by activating MPK4 via an HSP90 interaction (Cui *et al*., 2010). Results from these and similar studies are exemplary of an evolutionary ‘zigzag’ arms race between the host and pathogen (Jones and Dangl, 2006).

Our previous study pointed to a role for Ins*P*_6_ biosynthesis in maintaining basal defence responses (Murphy *et al*., 2008). It was thus the aim of our study to understand better the underlying molecular mechanisms of this role in plant defence. In this study, we present new findings on the effects of mutating the *IPS3* and *IPK1* genes on defence responses to Pst in arabidopsis. Interestingly, low‐Ins*P*_6_ *ipk1* mutant plants can establish SAR but are compromised in flg22‐induced resistance to localized infection, reinforcing the idea that the ability to synthesize Ins*P*_6_ is important in the maintenance of basal resistance mechanisms such as PTI. However, *ipk1* mutant plants appear to be unaffected in several well‐studied PAMP‐triggered responses, including the oxidative burst and Ca^2+^ influx, expression of early and late PTI marker genes, and inhibition of seedling growth.

## 2 RESULTS

### 2.1 Knockdown of *IPS3* resulted in hypersusceptibility to virulent and avirulent Pst

In arabidopsis there is a family of three *IPS* genes. We previously described T‐DNA insertion mutants for *AtIPS1* and *AtIPS2* (Murphy *et al*., 2008), designated here as *ips1* and *ips2.* In this work we identified an *AtIPS3* mutant (corresponding to locus At5g10170) from the Salk Institute Genome Analysis Laboratory population of mapped insertions (Alonso *et al*., 2003: SALK_ 097,807) from a segregating population. Transcript levels for *IPS1*, *IPS2*, and *IPS3* were compared in the three *ips* mutants and to the transformation control by reverse transcription coupled to the quantitative polymerase chain reaction (RT‐qPCR). IPS1 expression was enhanced in *ips2* but unaffected in *ips3* and *ipk1* (Figure S2). IPS2 expression was enhanced in *ips1* and *ips3* and was unaffected in *ipk1* while IPS3 expression was enhanced in *ips2* but unaffected in *ips1* and *ipk1* (Figure S2). This indicated that the *ips3* T‐DNA insertion mutant could be used as a loss‐of‐function mutant in pathology experiments.

**Figure 1:**
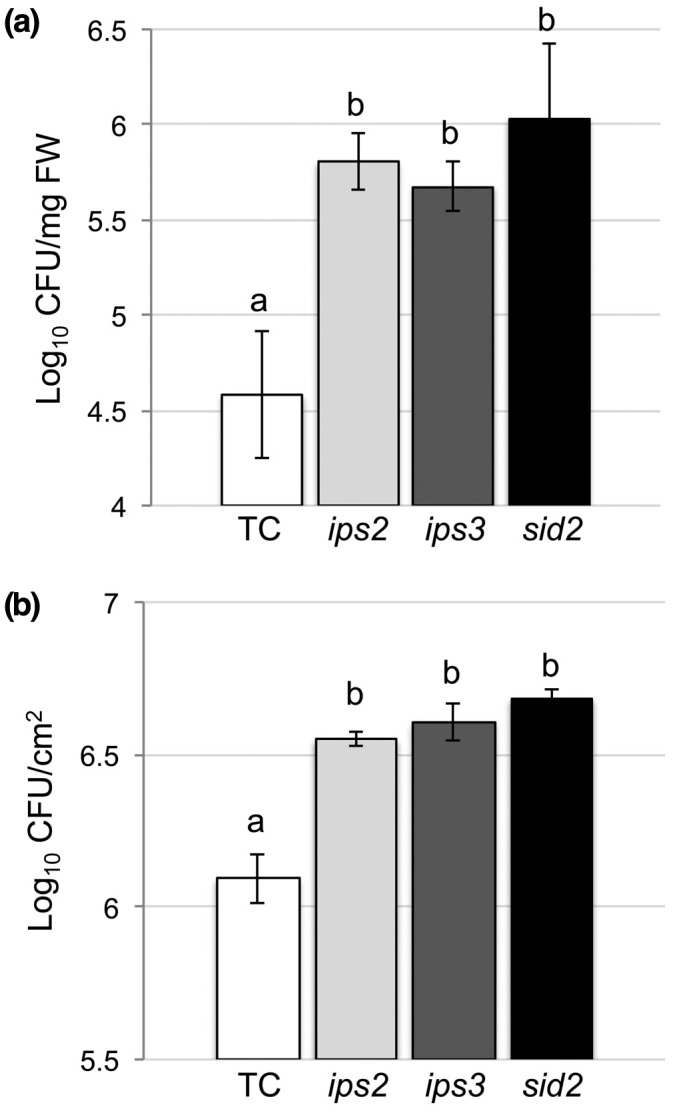
The arabidopsis *ips3* mutant is hypersusceptible to virulent *Pseudomonas syringae* pv. *tomato* (Pst). Leaf tissues from arabidopsis plants were infiltrated with a suspension of virulent Pst (10^5^ cfu/ml) and samples harvested at 2 days post‐inoculation, followed by serial dilution to determine accumulation of viable bacteria. (a) CFU in leaf samples (two leaves per plant, *n* = 4 plants) normalized to plant tissue fresh weight. (b) CFU in leaf samples (two leaves per plant, *n* = 4–5 plants) normalized to leaf disc area. Values not sharing the same lowercase letter are significantly different (*p* < .05: ANOVA and Tukey's test). Error bars represent standard error around the mean (*SEM*)

**Figure 2:**
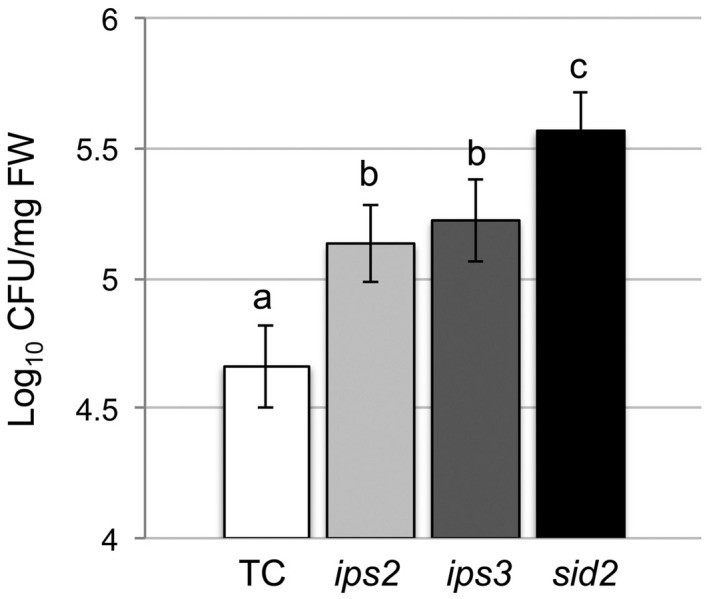
The arabidopsis *ips3* mutant is hypersusceptible to avirulent *Pseudomonas syringae* pv. *tomato* (Pst) expressing *AvrB*. Arabidopsis plants were infiltrated with a suspension of avirulent Pst (5 × 10^5^ cfu/ml). At 2 days post‐inoculation, leaf samples were harvested according to fresh weight (mg) for bacterial serial dilution assays. Results were pooled from three experiments for statistical analysis (three leaves per plant, *n* = 14–17 plants). Values not sharing the same lowercase letter are significantly different (*p* < .05: ANOVA and Tukey's test). Error bars represent *SEM*

We examined the responses of *ips3* plants to virulent Pst and found that they were significantly more susceptible to the bacterial pathogen than transformation control plants (Figure 1). The susceptibility of *ips3* to Pst was similar to that of *ips2*, previously shown to be hypersusceptible to Pst (Murphy *et al*., 2008), and the *sid2* mutant (Wildermuth *et al*., 2001; Macaulay *et al*., 2017), which is impaired in salicylic acid (SA) biosynthesis and highly susceptible to pathogen attack (Figure 1). As would be anticipated, plants carrying both the *ips2* and *ips3* mutations showed increased susceptibility to virulent Pst (Figure S3). Assessing bacterial growth in the mutant and control plants in relation to infected tissue fresh weight (Figure 1a) or according to infected leaf area (Figure 1b) produced comparable results. Mutant *ips3* plants also showed increased susceptibility to avirulent (harbouring the *AvrB* effector gene) and virulent Pst cells, as did *ips2* and *sid2* plants (Figures 2 and S3).

### 2.2 Mutations affecting Ins*P*_6_ biosynthesis did not inhibit the establishment of SAR

**Figure 3:**
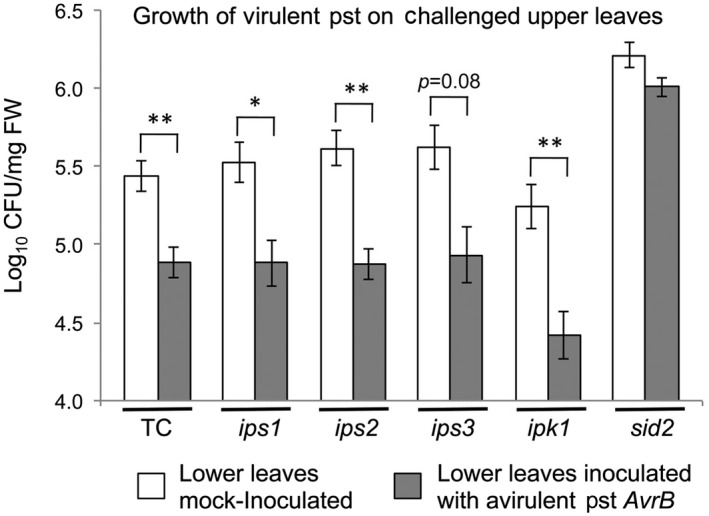
Systemic acquired resistance is not dependent on Ins*P*_6_ biosynthesis. Lower, fully expanded leaves of arabidopsis plants were initially inoculated with a mock solution (10 mM MgCl_2_) or a 5 × 10^5^ cfu/ml suspension in 10 mM MgCl_2_ of avirulent *Pseudomonas syringae* pv. *tomato* (Pst) harbouring a plasmid encoding the AvrB effector to stimulate systemic acquired resistance. Upper, noninoculated leaves were challenge‐inoculated with virulent Pst cells 4 days later. Two days following challenge, leaf samples were harvested for serial dilution assays. Results were pooled from five independent experiments for statistical analysis (two leaves per plant, *n* = 14–33 plants). Asterisks denote statistically significant differences between treatments of the respective genotypes (unequal variances with Welch's ANOVA, and Games–Howell post hoc test, *p < .05 and **p < .01)

SAR can be induced in plants following inoculation with an avirulent pathogen and triggering of ETI/the hypersensitive response. As disruption of Ins*P*_8_ interaction with the COI‐JAZ receptor diminishes JA‐regulated defences (Laha *et al*., 2015) and JA signalling is important in long‐distance signalling for SAR (Truman *et al*., 2007), it was possible that lesions in Ins*P*_6_ biosynthesis may affect SAR. To determine if Ins*P*_6_ biosynthesis is vital for establishment of SAR, *ips1*, *ips2 ips3*, and *ipk1* mutant plants were inoculated on lower leaves either with Pst cells harbouring a plasmid expressing the *AvrB* gene or with mock inoculum (10 mM MgCl_2_). Four days after inoculating the lower leaves, upper leaves were challenge inoculated with a suspension of virulent Pst and bacterial growth in these challenged leaves was determined 2 days later (Figure 3). Arabidopsis plants of the Col‐0 accession possess the R gene *RPM1* that mediates indirect recognition of the bacterial effector AvrB and induces ETI (Boyes *et al*., 1998; Mackey *et al*., 2002). Non‐mutant (transformation control plants) exhibited induction of SAR, as indicated by decreased bacterial growth in the upper, Pst‐challenged leaves of plants that had previously been inoculated with cells of the avirulent bacterial strain (Figure 3). As expected, *sid2* mutant plants, which are compromised in their ability to synthesize the SAR‐inducing signal SA, did not exhibit SAR (Figure 3). All four of the Ins*P*_6_ biosynthetic mutants exhibited SAR, indicating that while inhibiting expression of *IPS2*, *IPS3*, and *IPK1* diminishes basal resistance at the site of inoculation (Figures 1 and 2), it has no effect on defensive systemic signalling following ETI and the consequent establishment of SAR.

### 2.3 A mutation affecting the final step of Ins*P*_6_ biosynthesis inhibited flg22‐induced immunity

**Figure 4:**
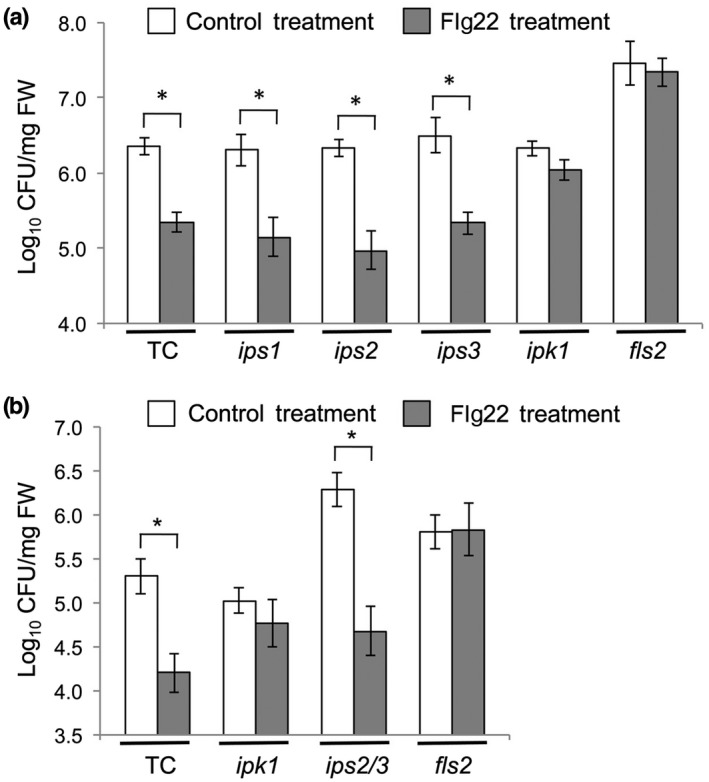
Local acquired resistance induced by flg22 was dependent on the *IPK1* gene. Leaves of arabidopsis plants were locally infiltrated with a control (water) treatment or a 1 μM flg22 solution and 1 day later the same leaves were challenge‐inoculated with virulent *Pseudomonas syringae* pv. *tomato* (Pst) (10^5^ cfu/ml). Leaf discs were sampled at 3 days post‐inoculationand leaf extracts serially diluted to determine bacterial titres. (a) Results for transformation control (TC) plants and plants of the mutant lines *ips1*, *ips2*, *ips3*, *ipk1* and the flg22‐insensitive mutant, *fls2*. (b) Data from additional experiments that included plants of the double *ips2/3* mutant line. Each panel shows results pooled from three independent experiments for statistical analysis (two leaves per plant, *n* = 9–18 plants). Asterisks denote significant differences between the indicated samples (unequal variances with Welch's ANOVA, Games–Howell post hoc test, *p* < .05)

The best‐studied basal immunity mechanism is PTI, therefore we investigated the responses of mutants in Ins*P*_6_ biosynthesis to one of the best studied experimental PAMPS, the synthetic oligopeptide flg22. Arabidopsis plants were infiltrated with water (control treatment) or a solution of flg22 1 day prior to a challenge with virulent Pst. As expected, transformation control plants exhibited flg22‐induced resistance to virulent Pst, whereas plants carrying a mutant allele for the flg22 receptor, FLS2, did not (Figure 4). The *ips1*, *ips2*, and *ips3* mutants all exhibited flg22‐induced resistance to Pst, indicating that PTI was not impaired in these mutants (Figure 4). However, *ipk1* mutant plants, like *fls2* mutants, did not respond to flg22 (Figure 4). Thus, PTI is inhibited by mutation of *IPK1* but not by mutation of *IPS* genes, suggestive that Ins*P*_6_ is an important factor underpinning PTI.

### 2.4 Double *ips2/3* mutant plants were compromised in basal resistance but still exhibited flg22‐induced resistance

The *ips2*, *ips3*, and *ips2/3* mutant plants were, like *ipk1*, hypersusceptible to Pst infection (Figure S3), but responded differently from *ipk1* plants to flg22. *IPS2* transcript accumulation was enhanced in *ips3* and *IPS3* expression was enhanced in *ips2*, suggesting that some regulatory cross‐talk occurs between the systems controlling *IPS2* and *IPS3* gene regulation (Figure S2), but both IPS2 and IPS3 expression was knocked down in the double *ips2/3* mutant (Figure S4). We therefore tested the ability of the *ips2/3* double mutant to exhibit flg22‐induced resistance to Pst (Figure 4b). The *ips2/3* double mutant was hypersusceptible to Pst (Figure S3), but it was not impaired in its ability to exhibit flg22‐induced resistance (Figure 4b). Thus, although *ips2, ips3*, and *ipk1* were compromised in their ability to resist Pst, only the *ipk1* mutant was specifically compromised in flg22‐induced resistance.

### 2.5 The expression of flg22‐induced PTI marker genes was not affected in *ipk1* mutant plants

**Figure 5:**
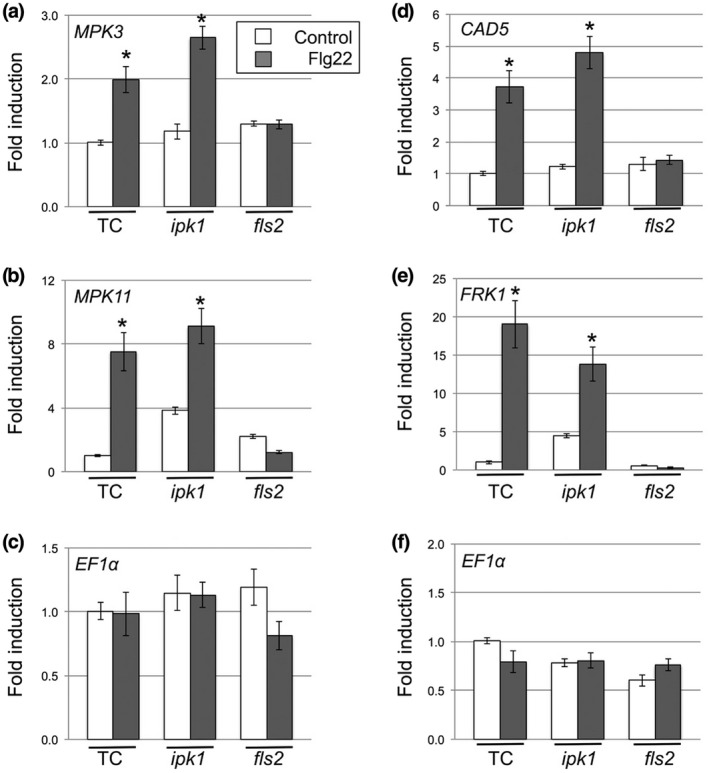
Mutation of the *IPK1* gene did not inhibit flg22‐induced gene expression. Arabidopsis leaves (from transformation control TC, *ipk1*, and *fls2* plants) were infiltrated with 1 μM flg22 or control solution (water) and sampled 30 min after treatment (for assay of MPK3 and MPK11) (a, b) or 3 hr after treatment for CAD5 and FRK1 (d, e). In each experiment, leaves from at least five plants per genotype were pooled for RNA extraction and RT‐qPCR with appropriate primers. Changes in transcript accumulation were normalized to the housekeeping gene *GAPDH* but accumulation of *EF1a* was used as an additional internal control (c, f). Error bars represent *SEM*. Asterisks denote significant differences from control treatments (ANOVA, Tukey's post hoc test, *p < .05). Three independent experiments were carried out with similar results

To determine if Ins*P*_6_ influences expression of PTI‐related genes, we investigated the steady‐state transcript levels of early (*MPK3* and *MPK11*: maximal expression 30 min after treatment) and late (*CAD5* and *FRK1*: maximal expression 3 hr after treatment) flg22‐dependent genes in the *ipk1* mutant. Expression of *MPK3* in transformation control plants increased by 2‐fold following flg22 treatment, and there was a similar (2.5‐fold) increase in *ipk1* mutant plants (Figure 5a). Expression of *MPK11* increased 7‐fold and 9‐fold in transformation control plants and *ipk1* mutant plants, respectively (Figure 5b). Overall, the up‐regulation of *MPK3* and *MPK11* can be specifically attributed to flg22 treatment because the negative control, *fls2* mutant, did not exhibit a transcriptional response to flg22 treatment (Figure 5).

Expression of late‐expressed flg22‐induced genes, *CAD5* and *FRK1*, was unaffected in *ipk1* mutant plants (Figure 5d,e). As expected, the *fls2* mutant did not exhibit any increase in the expression of flg22‐specific late marker genes (Figure 5d,e). Accumulation of the transcript for *EF1a* was used at both time points as an additional control and check for stability of expression of *GAPDH* (Figure 5c,f). Control gene expression was stable across treatments and genotypes, demonstrating that up‐regulation of flg22‐induced marker genes detected in *ipk1* mutant plants can be specifically attributed to elicitation by the flg22 oligopeptide.

### 2.6 The flg22‐induced calcium influx, oxidative burst, and root growth inhibition were not affected in the *ipk1* mutant

Recognition of PAMPs such as flg22 during PTI is associated with an influx of Ca^2+^ ions into the cell, which triggers production of superoxide anions catalysed by NADPH oxidase (Macho and Zipfel, 2014). As *ipk1*, *ips2*, and *ips3* mutants were compromised in basal resistance and *ipk1* was also compromised in flg22‐induced resistance, we examined the ability of mutants to exhibit the flg22‐triggered Ca^2+^ ion influx and oxidative burst using, respectively, an aequorin transgene and a luminol‐based assay. To examine whether plants depleted in Ins*P*_6_ are affected with respect to the flg22‐triggered Ca^2+^ influx into the cytosol calcium influx signals, we generated transgenic plants expressing the Ca^2+^ reporter aequorin in the *ipk1* mutant background (Dodd *et al*., 2010; Cheval *et al*., 2013). When treated with flg22, aequorin‐expressing transgenic *ipk1* seedlings exhibited a similar influx of Ca^2+^ to aequorin‐transgenic arabidopsis possessing wild‐type *IPK1* alleles, indicating that Ins*P*_6_ is not a key factor in this PTI‐associated phenomenon (Figure S5). Using a luminol assay to detect reactive oxygen species generation, leaf discs from *ips1*, *ips2*, *ips3*, and *ipk1* mutant plants responded to flg22 with an oxidative burst of the same magnitude and in the same time frame as control plants (Figure S6).

Seedling growth inhibition is another well‐characterized response of arabidopsis plants to flg22 (Gómez‐Gómez *et al*., 1999). When treated with varying concentrations of flg22, control plants displayed a decrease in root growth proportional to flg22 concentration (Figure S7) while the *fls2* mutant (included as a control) did not exhibit flg22‐triggered growth inhibition. The *ipk1* mutant plants also displayed flg22‐triggered inhibition of seedling root growth (Figure S7), indicating that the *IPK1* gene is not involved in the molecular processes underlying growth inhibition induced by the PAMP flg22.

### 2.7 Hypersusceptibility to virulent Pst in *ips2*, *ips3*, and *ipk1* mutant plants was lost after wounding

It was noted during experiments in which mock inoculation (infiltration of a control solution) was carried out on a lower leaf that subsequent bacterial growth on an upper leaf of the *ips2*, *ips3*, and *ipk1* mutant plants was similar to the transformation control plants (Figures 3 and 4). This suggested that the mock inoculation rescued the impaired defence responses in the hypersusceptible Ins*P*_6_ biosynthetic mutants. Experiments were then carried out to determine whether the cause of resistance induced by the mock treatment was due to wounding (as a result of the infiltration method, pressing a syringe against the tissue) or the infiltration of a solution into the apoplast (flooding of the intercellular space, potentially causing anoxia). The enhanced susceptibility to Pst exhibited by Ins*P*_6_ biosynthesis mutants was lost after water infiltration (Figure S8) or wounding (Figure S9). These data indicate that the resistance induced by mock treatment is due to wounding and that wound‐induced resistance is not impaired in the Ins*P*_6_ biosynthesis pathway mutants.

## 3 DISCUSSION

Previous results showed that Ins*P*_6_ biosynthesis is required for the maintenance of basal resistance against viral, bacterial, and fungal pathogens in arabidopsis and potato (Murphy *et al*., 2008), and resistance to cyst nematode infestation (Jain *et al*., 2015). Interestingly, a recent study highlighted the importance of a higher‐order inositol polyphosphate, Ins*P*_8_, in plant resistance to chewing herbivores (Laha *et al*., 2015). Ins*P*_8_, like Ins*P*_6_, is dependent on the enzymes IPS and IPK for its biosynthesis (Figure S1). To investigate the mechanism(s) underlying the role of Ins*P*_6_ in pathogen resistance, we analysed the responses of *ips3* mutant plants to microbial infection and dissected the various PAMP‐triggered responses in *ipk1* mutant arabidopsis plants. We found that *ips3* mutant plants were as hypersusceptible to virulent or avirulent Pst as *ips2* mutant plants, which implicates IPS2 and IPS3 in synthesis of distinct pools of Ins*P*_6_ that are required for effective basal defence. Furthermore, the vascular and hydathode‐specific expression of the *IPS2* and *IPS3* genes in arabidopsis (Donahue *et al*., 2010) strongly suggests a critical involvement of Ins*P*_6_ biosynthesis in specific cells and tissues that can act as barriers to pathogen ingress (hydathodes) or dissemination through the host (vasculature). Hydathodes are open pores on the leaf margin that provide an entry point for bacterial pathogens such as Pst into the apoplast (Rufián *et al*., 2018). Elevated levels of defence‐related gene expression in cells surrounding hydathodes has been previously reported indicating a role of localized constitutive defence around these vulnerable openings (Macaulay *et al*., 2017; van den Burg *et al*., 2010).

Our finding that *ipk1* mutant plants could exhibit SAR but not flg22‐induced resistance suggests a differentiation in the requirement for Ins*P*_6_ biosynthesis between these two plant defence responses. We showed that basal levels of SA were not diminished in *ips2* or *ipk1* and that SA biosynthesis increased in response to inoculation with avirulent Pst in a similar manner to transformation control plants (Murphy *et al*., 2008). Taken together, our previous data and new findings demonstrate that the hypersusceptible *ipk1* mutants are not impaired in SA‐mediated defensive signalling, nor in the ability to express SAR, but that normal synthesis of Ins*P*_6_ is essential for PTI. However, an increase in resistance to Pst induced by injection of air or water into the apoplast (which we presume to be wound or stress induced: Figures S8 and S9) appears to be a form of defence that is distinct from PTI and not dependent on Ins*P*_6_ biosynthesis.

The degree of impairment of flg22‐induced resistance in plants carrying a mutation in *IPK1* was similar to that in plants with a mutation in *FLS2*. However, *ipk1* mutant plants were not affected in a number of early flg22‐dependent responses, including the rapid oxidative burst, influx of Ca^2+^ ions, and the up‐regulation of flg22‐responsive transcripts. Our work contradicts certain previous findings on the effects of the *ipk1* mutation on flg22‐elicited gene expression. We found that in plants grown in soil under a normal day/night regime flg22‐induced transcripts were up‐regulated to a similar extent in soil‐grown *ipk1* mutant and control (*IPK1*) plants but that, nevertheless, flg22‐induced resistance to Pst was severely diminished in *ipk1* mutant plants. However, Ma *et al*. (2017) found that in *ipk1* mutant plants grown hydroponically and under continuous illumination, flg22‐induced transcriptional responses were diminished, although not abolished. The differences between these studies probably hinge on differences in growth conditions with, perhaps, the most important effect being photoperiod. Continuous light conditions disrupt the circadian system of the plant. This vital internal clock system regulates expression of defence‐related transcripts (Robertson *et al*., 2009) and responses of plants to infection (Genoud *et al*., 2002; Handford and Carr, 2007; Palukaitis *et al*., 2013).

Our results with *ipk1* mutants suggest that the increased local resistance to Pst induced by flg22 is not dependent on the transcriptional up‐regulation of *MAPK*s, but still depends on Ins*P*_6_ biosynthesis. Possible explanations may include redundancy in downstream defensive signalling, or that the system is not absolutely dependent on transcriptional changes in expression of these factors or that post‐transcriptional effects such as protein phosphorylation are more important than changes in the steady‐state accumulation of these proteins. Flg22‐induced resistance is also independent of signalling mediated by SA, JA, and ethylene (Zipfel *et al*., 2004). It was noted during this study that injecting air or water into the apoplast 24 hr before challenge with Pst induced an increase in resistance to this pathogen, presumably a form of wounding‐induced resistance. This form of resistance was not affected by the *ipk1* mutation, which suggests that basal resistance involves multiple signalling pathways, not all of which require Ins*P*_6_ biosynthesis for operation. In conclusion, it appears that Ins*P*_6_ biosynthesis is required for flg22‐triggered resistance but it remains unknown whether resistance elicitation by other PAMPs also depends on this. However, based on the broad range of pathogens that are able to overcome basal resistance in Ins*P*_6_‐depleted plants (Murphy *et al*., 2008), this would seem likely.

The hypersusceptibility to Pst of *ips2* and *ips3* single mutant plants indicates that both of the isoenzymes IPS2 and IPS3 are necessary for basal resistance to pathogen attack, that is, they are not redundant factors in maintenance of this form of defence. The double *ips2/3* mutant was also hypersusceptible to Pst infection and, like the single *ips2* and *ips3* mutants, was not impaired in flg22‐induced resistance. In contrast, plants carrying a mutation in the *IPK1* gene were compromised in basal resistance and in flg22‐stimulated resistance to Pst. Several possibilities might account for the difference between the *ips* and *ipk1* mutants. The set of proteins contributing to the pools of synthesized Ins*P*_6_ might be partitioned and differ for basal resistance or flg22‐induced resistance. In our experiments, we investigated the defence responses of mutants affected in steps in the Ins*P*_6_ lipid‐independent pathway and found, for example, that *ipk1* mutants were not compromised in flg22‐induced Ca^2+^ influx into the cytosol. However, it has been shown that plants depleted in lipid‐derived Ins*P*_3_ have a diminished flg22‐induced Ca^2+^ influx response (Ma *et al*., 2017). It is possible, therefore, that both the lipid‐independent and the lipid‐dependent Ins*P*_6_ biosynthetic pathway (Figure S1) might each be able to contribute to distinct Ins*P*_6_ pools needed for different aspects of basal resistance and flg22‐induced responses.

In conclusion, our work confirms that normal Ins*P*_6_ biosynthesis is important in maintaining basal defences against pathogens and also shows that it contributes to more than one defensive mechanism. In creating low‐phytate crops, it may be wise not to inhibit synthesis of Ins*P*_6_ in all tissues but to do so only in those parts that are to be consumed in the human diet or processed as animal feeds, an approach shown to be feasible in, for example, soybean where levels of this metabolite were selectively decreased in the seeds (Kumar *et al*., 2019).

## 4 EXPERIMENTAL PROCEDURES

### 4.1 Plant material and growth conditions

*A. thaliana* accession Col‐0 plants were grown under short‐ or long‐day conditions as described below. Seeds for *Atipk1*, *Atips1*, and *Atips2* mutants were from pools previously confirmed to be homozygous for the T‐DNA insertion (Murphy *et al*., 2008). *AtIPS3* corresponds to locus At5g10170. Seeds for *Atips3* (SALK_ 097,807) were obtained from the Nottingham Arabidopsis Stock Centre (NASC, http://www.arabidopsis.info), and a homozygous line for the T‐DNA insertion was obtained after verification by PCR genotyping and sequencing. Transformation control (indicated by TC in figures) plants do not have the T‐DNA insertion and were selected from the original segregating T‐DNA mutant populations to serve as controls in the experiments, as previously described by Murphy *et al*. (2008).

For infection, oxidative burst, and flg22‐induced transcription experiments, seeds were sown in a 4:1 compost:sand mixture and stratified for 2 days at 4 °C. Seeds were germinated and grown under short‐day conditions (8 hr light/16 hr dark cycles, 22 °C, 60% relative humidity, and 200 μmol⋅ m^–^^2^⋅s^–^^1^ photosynthetically active radiation) in a Conviron growth room. For inhibition of seedling growth experiments, seeds were surface sterilized and stratified for 2 days at 4 °C on 0.5 × Murashige and Skoog (MS) basal salts, 1% (wt/vol) agar. Seeds were germinated and grown under long‐day conditions (16 hr light/8 hr dark cycles, 21 °C, and 200 μmol⋅m^–^^2^⋅s^–1^ photosynthetically active radiation). For Ca^2+^ influx experiments, seedlings were grown in liquid 0.5 × MS basal salts medium.

### 4.2 Bacterial procedures

Virulent *P. syringae* pv. *tomato* (Pst) DC3000 was maintained on Luria–Bertani (LB) agar (Sambrook *et al*., 1989) containing 50 µg/ml rifampicin at 25 °C and avirulent Pst carrying the *AvrB* gene (Pst AvrB) was maintained in the same manner with additional antibiotic selection with 50 µg/ml kanamycin (Murphy *et al*., 2008). Bacterial inoculum was prepared by streaking out bacterial colonies 1 day prior to the experiment (Tornero and Dangl, 2001). On the day of the experiment, the colonies were initially suspended in 5 ml 10 mM MgCl_2_ and diluted to achieve 10^5^ cells/ml for all infiltration experiments (unless otherwise stated). Leaves of 4‐week‐old arabidopsis plants (that had been grown under short‐day conditions) were inoculated using a needleless 1 ml syringe (Klement, 1963). Two days post‐inoculation (dpi), unless otherwise stated, leaf samples were taken to determine bacterial growth titres. Plant tissue samples were taken by recording fresh weight (mg) per leaf or taking 2‐mm diameter leaf discs with a cork borer. Samples were homogenized in 10 mM MgCl_2_, serially diluted and plated on LB agar to determine the titre of viable bacterial cells, using counts of colony‐forming units (cfu).

### 4.3 Testing for establishment of SAR

A method modified from Cameron *et al*. (1999) was used to test for SAR induction in noninoculated leaves. Bacterial inoculum was prepared as described above. Lower leaves of arabidopsis plants were infiltrated with either 10 mM MgCl_2_ (mock inoculum) or a suspension of avirulent Pst AvrB at 5 × 10^5^ cells/ml. Four days later, upper leaves of the same plants were challenge inoculated with a suspension of virulent Pst at 5 × 10^5^ cells/ml. Three days after the challenge inoculations, unless otherwise stated, leaf samples were taken and bacterial titres determined as described above.

### 4.4 Testing for flg22‐induced local acquired resistance

To test for flg22‐induced local acquired resistance, a modified method based on Zipfel *et al*. (2004) was used. Arabidopsis leaves were treated with either a mock treatment (water) or 1 μM flg22 24 hr before inoculation with a suspension of virulent Pst (10^5^ cells/ml). At 3 dpi, discs of tissue were harvested from the inoculated leaves and bacterial titres determined as described above.

### 4.5 Oxidative burst assay

A luminol–horseradish peroxidase (HRP) assay adapted from methods previously described (Whitehead *et al*., 1983; Keppler *et al*., 1989) was used to measure oxidative bursts in arabidopsis. Leaf discs (4.5 mm diameter) from 4‐week‐old arabidopsis plants grown under short‐day conditions were placed in opaque white 96‐well microtitre plates with 150 μl water per well overnight. The release of reactive oxygen species was measured using a luminol‐dependent assay by replacing the water with 100 μl of 100 μM luminol (Sigma) containing 10 μg/ml HRP (250 units/mg solid: Sigma) and the PAMP flg22 (100 nM) (Whitehead *et al*., 1983; Keppler *et al*., 1989; Kunze *et al*., 2004). Luminescence was measured immediately using a FLUOstar OPTIMA multimode microplate reader (BMG Labtech) for 20 min.

### 4.6 Seedling growth inhibition

Seedlings were grown vertically on 0.5 × MS agar plates containing varying concentrations of flg22 (10 nM, 1 μM, 2.5 μM). Root growth inhibition was assessed at 3 and 7 days after germination using ImageJ v. 1.43u (National Institutes of Health, Bethesda MD) to measure the lengths of primary roots from scanned images.

### 4.7 Reverse transcription‐coupled quantitative PCR and analysis of gene expression

Seeds were grown under short‐day conditions, and leaves from 4‐week‐old plants were infiltrated with 1 μM flg22 or water. At 30 min and 3 hr following treatment, infiltrated leaves were harvested and flash‐frozen in liquid nitrogen. Total RNA for reverse transcription‐coupled quantitative PCR (RT‐qPCR) analysis was extracted using TRIzol reagent (Invitrogen) according to the manufacturer's protocol, followed by lithium chloride precipitation, phenol–chloroform extraction, and ethanol precipitation (Berry *et al*., 1985). Total RNA samples were treated with TURBO‐DNase (Ambion) according to the manufacturer's protocol. Complementary DNA (cDNA) was synthesized from 0.5 μg total RNA using GoScript Reverse Transcription System (Promega) with oligo‐dT_15_ primers following the manufacturer's instructions in the presence of RNaseOUT Recombinant Ribonuclease Inhibitor (Life Technologies). The cDNA was diluted 10‐fold and quantitative PCR (qPCR) was performed using SensiMix No ROX (Bioline) in 20 μl reactions, containing 250 nM each of the forward and reverse primers complementary to sequences of interest (Table S1). The transcripts for *glyceraldehyde‐3‐phosphate dehydrogenase* (*GAPDH*) and *elongation factor 1A* (*EF1α*) were used as internal standards (Westwood *et al*., 2013). PCRs were conducted in triplicate using the CFX96 Touch Real‐Time PCR Detection System (Bio‐Rad). Data were analysed using LinRegPCR v. 2014.8 (Hartfaal Centrum, the Netherlands) to calculate amplification efficiencies and *C*_t_ values. Expression levels of genes of interest relative to *GAPDH* and *EF1α* were calculated using the ΔΔ*C*_t_ method (Livak and Schmittgen, 2001; Ramakers *et al*., 2003).

## Supporting information


**FIGURE S1** Ins*P*_6_: its biosynthesis and turnover in plants

**FIGURE S2** Expression of (a)* IPS1*, (b) *IPS2*, and (c) *IPS3* was measured in *ips *and* ipk1 *mutant plants and compared to transformation control (TC) plants

**FIGURE S3** Arabidopsis *ips2/3* double mutant plants were hypersusceptible to virulent *Pseudomonas syringae *pv. *tomato*

**FIGURE S4** Expression of *IPS1*, *IPS2*, and *IPS3* in *ips2/3 *double mutant compared to transformation control (TC) plants

**FIGURE S5** The flg22‐induced influx of Ca^2+ ^ions was not affected by mutation of the *IPK1* gene

**FIGURE S6** The flg22‐induced oxidative burst was not affected in Ins*P*_6_ biosynthetic mutants

**FIGURE S7** Flg22‐induced root growth inhibition was unaffected in the *ipk1* mutant

**FIGURE S8** Pretreatment with water infiltration induced resistance to *Pseudomonas syringae* in the normally hypersusceptible *ips2*, *ips3* and *ipk1* mutants

**FIGURE S9** Infiltration of leaves with air or water 1 day before challenge with* Pseudomonas syringae *induced resistance in nonmutant plants and *ipk1* mutants but not in* fls2 *mutant plants

**TABLE S1** List of primers used in this study

## Data Availability

The data that support the findings of this study are available from the corresponding author upon reasonable request.
