# Supplementary material for: Inositol hexakisphosphate biosynthesis underpins PAMP‐triggered immunity to Pseudomonas syringae pv. tomato in Arabidopsis thaliana but is dispensable for establishment of systemic acquired resistance
Source: Mol Plant Pathol. 2019 Dec 26;21(3):376–87. doi: 10.1111/mpp.12902 (PMC7036367; doi:10.1111/mpp.12902)
Supplement: Supplementary file 1 — FIGURE S1 InsP 6: its biosynthesis and turnover in plants [file MPP-21-376-s001.pdf]

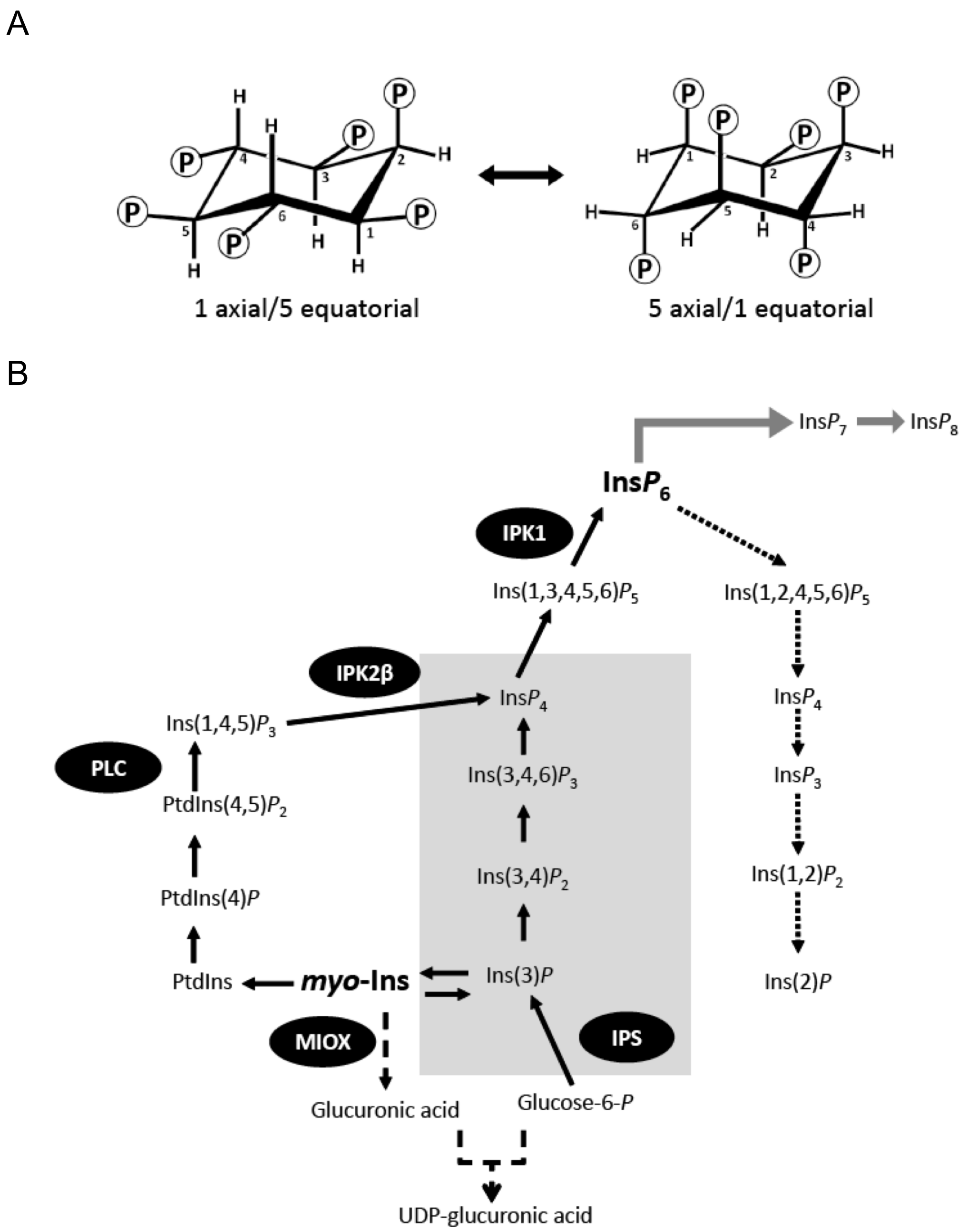

Fig S1. InsP_6: its biosynthesis and turnover in plants. Chemical structures of Ins*P*6 (A) at low pH (1 axial/5 equatorial) (left) and high pH (5 axial/1 equatorial) (right). Steps leading to Ins*P*6 biosynthesis (B) are designated by black arrows. The pathway originating from PI(4,5)*P*2 is lipid-dependent, whereas the lipid-independent pathway originating from Ins3*P* or *myo*-inositol (*myo*-Ins) is shaded in light grey. Degradation of InsP_6 by dephosphorylation is designated by dotted arrows, and steps leading to higher inositol pyrophosphates are marked by grey arrows. Steps towards UDP-glucuronic acid biosynthesis are denoted by dashed arrows. The following abbreviations are used: MIOX, *myo*-inositol oxygenase; IPS, inositol phosphate synthase; IPK1, inositol penta*kis*phosphate 2-kinase; IPK2, inositol polyphosphate kinase, and PLC, phospholipase C. Based on Brearley, C. A. and Hanke, D. E. (1996) Biochem. J, 31,:227–233; Kim, S. I. and Tai, T. H. (2011) Molec. Gen. Genom. 286,119–133; Hanke, D.E. *et al.* (2012) Biochem. J. 444, 601–609; Isbrandt, L.R. and Oertel, R.P. (1980) J. Amer. Chem. Soc. 102, 3144–3148., and Laha *et al.* (2015) Plant Cell, 27,1082–1097.
