# Supplementary material for: Inositol hexakisphosphate biosynthesis underpins PAMP‐triggered immunity to Pseudomonas syringae pv. tomato in Arabidopsis thaliana but is dispensable for establishment of systemic acquired resistance
Source: Mol Plant Pathol. 2019 Dec 26;21(3):376–87. doi: 10.1111/mpp.12902 (PMC7036367; doi:10.1111/mpp.12902)
Supplement: Supplementary file 2 — FIGURE S2 Expression of (a) IPS1, (b) IPS2, and (c) IPS3 was measured in ips and ipk1 mutant plants and compared to transformation control (TC) plants [file MPP-21-376-s002.pdf]

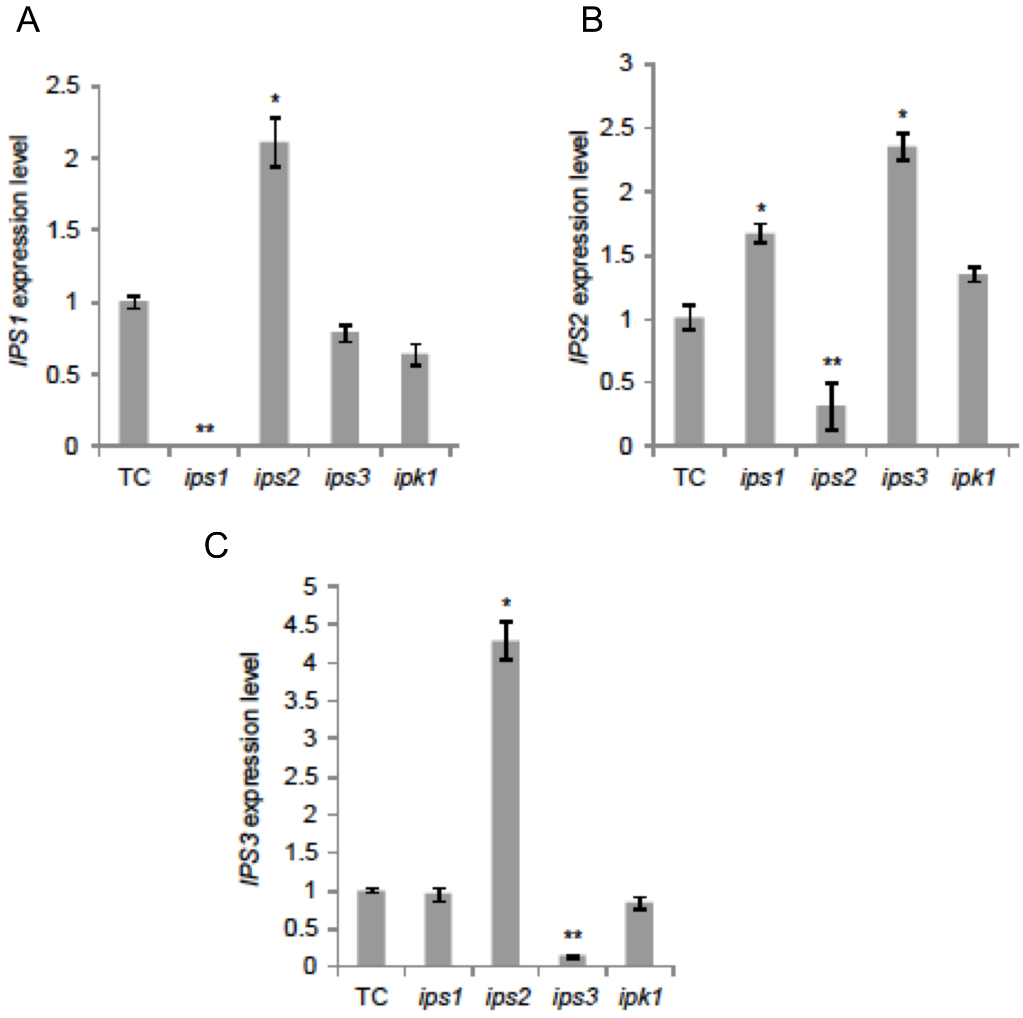

Fig. S2. Expression of (A) *IPS1*, (B) *IPS2* and (C) *IPS3* was measured in *ips* and *ipk1* mutant plants and compared to transformation control (TC) plants. Arabidopsis plants of the *ips, ipk1* or TC lines were grown under short day (8 h light/16 h dark) conditions, and vegetative tissue from these plants was extracted for RNA (pooled samples from 5 plants each), followed by DNase treatment and reverse transcription. Quantitative PCR was performed on cDNA for each genotype in technical quadruplicates, and expression levels of the target genes were normalized to the expression of the GAPDH housekeeping gene. Error bars represent SEM. One-way ANOVA, Tukey's post hoc test, ** indicating a significant decrease and * indicating a significant increase from the TC, $p < 0.05$.
