# Supplementary material for: Inositol hexakisphosphate biosynthesis underpins PAMP‐triggered immunity to Pseudomonas syringae pv. tomato in Arabidopsis thaliana but is dispensable for establishment of systemic acquired resistance
Source: Mol Plant Pathol. 2019 Dec 26;21(3):376–87. doi: 10.1111/mpp.12902 (PMC7036367; doi:10.1111/mpp.12902)
Supplement: Supplementary file 3 — FIGURE S3 Arabidopsis ips2/3 double mutant plants were hypersusceptible to virulent Pseudomonas syringae pv. tomato [file MPP-21-376-s003.pdf]

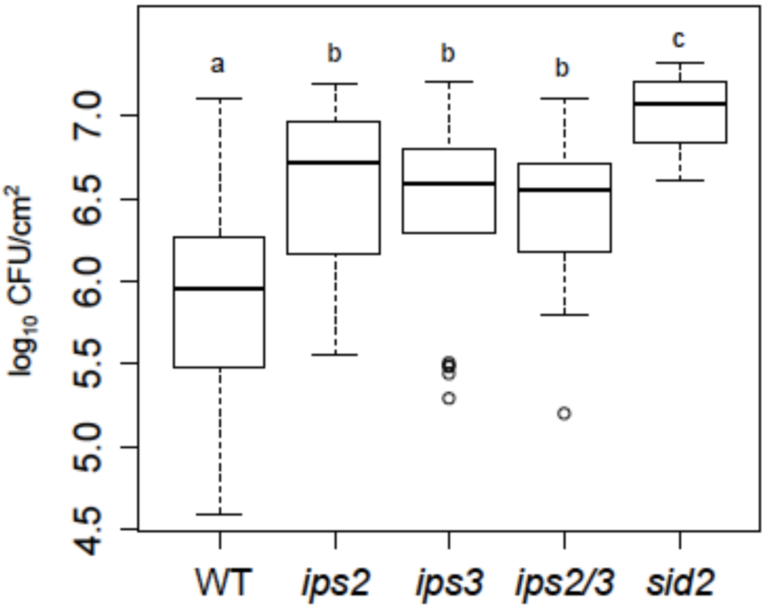

Fig. S3. Arabidopsis *ips2/3* double mutant plants are hypersusceptible to virulent *Pseudomonas syringae* pv. *tomato.* Leaves of wild type (WT) or mutant Col-0 Arabidopsis plants were infiltrated with a suspension of virulent Pst (10^5 CFU/ml). At two days post inoculation, leaf discs were harvested for bacterial serial dilution assays. Results were pooled from four experiments for statistical analysis (two leaves per plant, n = 24 - 27 plants). Data not sharing the same letter are significantly different (unequal variances with Welch's ANOVA, and Games-Howell post hoc test, $p < 0.01$).
