# Supplementary material for: Inositol hexakisphosphate biosynthesis underpins PAMP‐triggered immunity to Pseudomonas syringae pv. tomato in Arabidopsis thaliana but is dispensable for establishment of systemic acquired resistance
Source: Mol Plant Pathol. 2019 Dec 26;21(3):376–87. doi: 10.1111/mpp.12902 (PMC7036367; doi:10.1111/mpp.12902)
Supplement: Supplementary file 4 — FIGURE S4 Expression of IPS1, IPS2, and IPS3 in ips2/3 double mutant compared to transformation control (TC) plants [file MPP-21-376-s004.pdf]

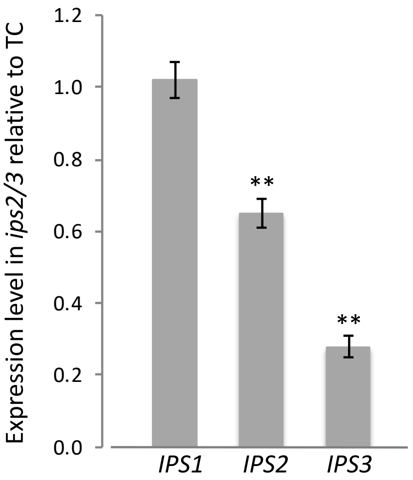

Fig. S4. Expression of *IPS1, IPS2 and IPS3* was measured in the arabidopsis double mutant *ips2/3* and expressed relative to the transformation control (TC) plant. Arabidopsis plants of the *ips 2/3, or* TC lines were grown under short day (8 h light/16 h dark) conditions, and vegetative tissue from these plants was extracted for RNA (pooled samples from 5 plants each), followed by Dnase treatment and reverse transcription. Quantitative PCR was performed on cDNA from ips2/3 in technical quadruplicates, and expression levels of the target genes were normalized to the expression of the GAPDH housekeeping gene. Error bars represent SEM. One-way ANOVA, Tukey's post hoc test, ** indicating a significant decrease from the TC, $p < 0.05$.
