# Supplementary material for: Inositol hexakisphosphate biosynthesis underpins PAMP‐triggered immunity to Pseudomonas syringae pv. tomato in Arabidopsis thaliana but is dispensable for establishment of systemic acquired resistance
Source: Mol Plant Pathol. 2019 Dec 26;21(3):376–87. doi: 10.1111/mpp.12902 (PMC7036367; doi:10.1111/mpp.12902)
Supplement: Supplementary file 5 — FIGURE S5 The flg22‐induced influx of Ca2+ ions was not affected by mutation of the IPK1 gene [file MPP-21-376-s005.pdf]

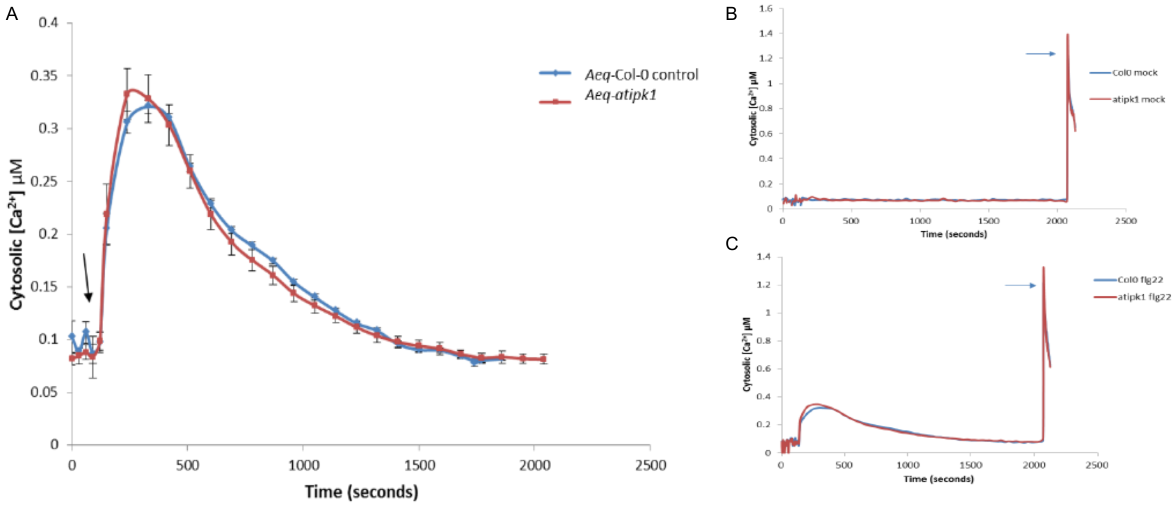

Fig. S5. The flg22-induced influx of Ca^{2+} ions is not affected by mutation of the *IPK1* gene. A transgene encoding the Ca^{2+} reporter protein aequorin was introduced into plants of the wild-type Col-0 Arabidopsis background (*IPK1*) and into the *ipk1* mutant background. (A) Addition of 1 μM flg22 (arrow) induced aequorin fluorescence in both plant backgrounds and the magnitude and kinetics of fluorescence induction were identical, indicating that $InsP_6$ is not required for flg22 Ca^{2+} influx. Data shown are from n = 8-10 plants per line. The experiment was repeated three times with similar results and similar results were obtained from two independent aequorin-expressing lines (*aeq*-Col0 control A5 and A8 and *aeq-atipk1* A7 and D1). Bars represent SEM. Data from the mutant were determined to be not statistically significant from the Col-0 control using Student's *t*-test. (B) Mock (water) treatment of aequorin transgenic Col-0 (*IPK1*) and *ipk1* background seedlings, where the average values of 6 seedlings per genotype are plotted. Maximum Ca^{2+} was discharged with 2M $CaCl_2$ at 35 minutes after the start of the experiment. Panel (C) shows the same trace from panel A with the 2M $CaCl_2$ discharge shown. Blue arrows indicate the peak of the Col0 control cytosolic [Ca2+] which is obscured in panels B and C by the red *atipk1* line.
