# Supplementary material for: Inositol hexakisphosphate biosynthesis underpins PAMP‐triggered immunity to Pseudomonas syringae pv. tomato in Arabidopsis thaliana but is dispensable for establishment of systemic acquired resistance
Source: Mol Plant Pathol. 2019 Dec 26;21(3):376–87. doi: 10.1111/mpp.12902 (PMC7036367; doi:10.1111/mpp.12902)
Supplement: Supplementary file 6 — FIGURE S6 The flg22‐induced oxidative burst was not affected in InsP 6 biosynthetic mutants [file MPP-21-376-s006.pdf]

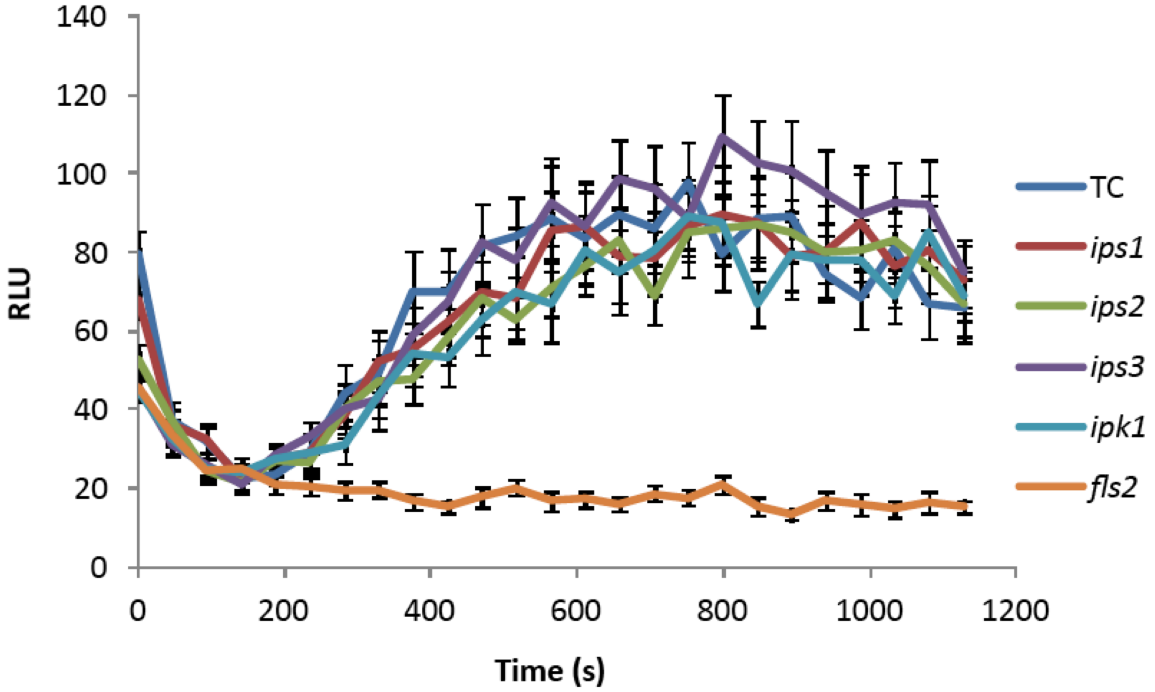

Fig. S6. The flg22-induced oxidative burst is not affected in $InsP_6$ biosynthetic mutants. Leaf discs from *ips* and *ipk1* mutants displayed similar oxidative burst characteristic of those from transformation control (TC) plants upon elicitation with 100 μM flg22. Relative luminescence units (RLU) were determined from leaf discs of the indicated mutant and TC plants using a luminol/horse radish peroxidase-based assay in a multi-mode microplate reader. Plants were grown in 8 h light/16 h dark cycles, and leaf discs from 4-week old plants were used (n = 16 plants). Error bars represent SEM.
