# Supplementary material for: Inositol hexakisphosphate biosynthesis underpins PAMP‐triggered immunity to Pseudomonas syringae pv. tomato in Arabidopsis thaliana but is dispensable for establishment of systemic acquired resistance
Source: Mol Plant Pathol. 2019 Dec 26;21(3):376–87. doi: 10.1111/mpp.12902 (PMC7036367; doi:10.1111/mpp.12902)
Supplement: Supplementary file 7 — FIGURE S7 Flg22‐induced root growth inhibition was unaffected in the ipk1 mutant [file MPP-21-376-s007.pdf]

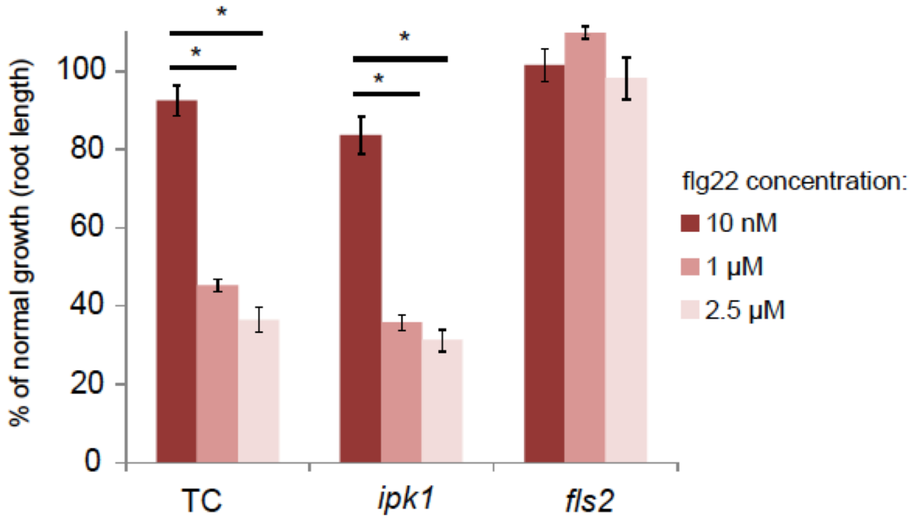

Fig. S7. Flg22-induced root growth inhibition was unaffected in the *ipk1* mutant. The *fls2* mutant seedlings served as controls and as expected root growth was not inhibited by flg22 in these mutant plants. Root growth of seedlings cultivated on 0.5 x MS medium (control: 100%, all values normalised against this) and flg22 (10 nM, 1 µM, or 2.5 µM) was compared. Seedlings were grown at 16 h light/8 h dark cycles at 21°C, and 12 seedlings per genotype were used. Error bars represent SEM. Asterisks denote significant differences between the indicated pairs (one-way ANOVA, Tukey's post hoc test, $p < 0.05$).
