# Supplementary material for: Inositol hexakisphosphate biosynthesis underpins PAMP‐triggered immunity to Pseudomonas syringae pv. tomato in Arabidopsis thaliana but is dispensable for establishment of systemic acquired resistance
Source: Mol Plant Pathol. 2019 Dec 26;21(3):376–87. doi: 10.1111/mpp.12902 (PMC7036367; doi:10.1111/mpp.12902)
Supplement: Supplementary file 8 — FIGURE S8 Pretreatment with water infiltration induced resistance to Pseudomonas syringae in the normally hypersusceptible ips2, ips3 and ipk1 mutants [file MPP-21-376-s008.pdf]

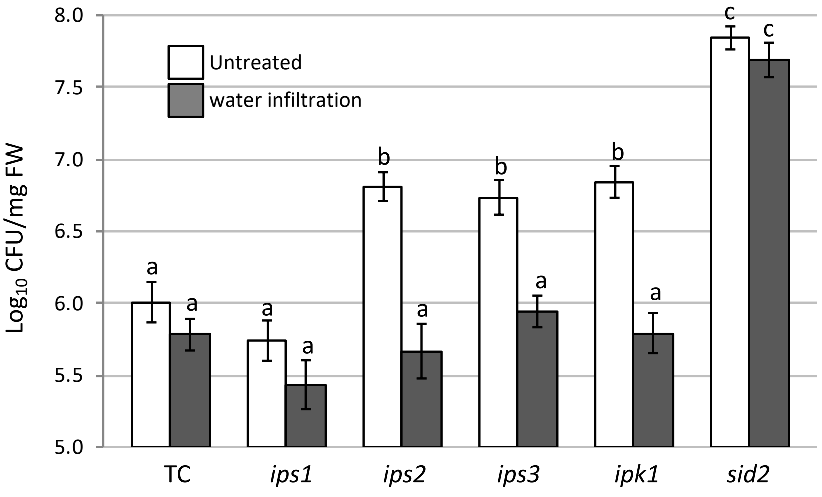

Fig. S8. Pre-treatment with water infiltration induces resistance to *Pseudomonas syringae* in the normally hypersusceptible low-InsP_6 mutant plants (*ips2*, *ips3*, and *ipk1*). Mutant and transformation control (TC) leaves were infiltrated with water using a syringe or left undisturbed and 24 h later, the same leaves were challenged with virulent Pst (10^5 CFU/ml). Leaf discs were sampled three days post inoculation and leaf extracts used for bacterial serial dilution assays. Results were pooled from two independent experiments for statistical analysis (2 leaves per plant, n = 9 - 10 plants). Although TC and *ips1* showed enhanced resistance after pre-treatment with water, this was not always statistically significant. One-way ANOVA, Tukey's post hoc test, genotypes not sharing the same letter are significantly different ($p < 0.05$).
