# Supplementary material for: Inositol hexakisphosphate biosynthesis underpins PAMP‐triggered immunity to Pseudomonas syringae pv. tomato in Arabidopsis thaliana but is dispensable for establishment of systemic acquired resistance
Source: Mol Plant Pathol. 2019 Dec 26;21(3):376–87. doi: 10.1111/mpp.12902 (PMC7036367; doi:10.1111/mpp.12902)
Supplement: Supplementary file 9 — FIGURE S9 Infiltration of leaves with air or water 1 day before challenge with Pseudomonas syringae induced resistance in nonmutant plants and ipk1 mutants but not in fls2 mutant plants [file MPP-21-376-s009.pdf]

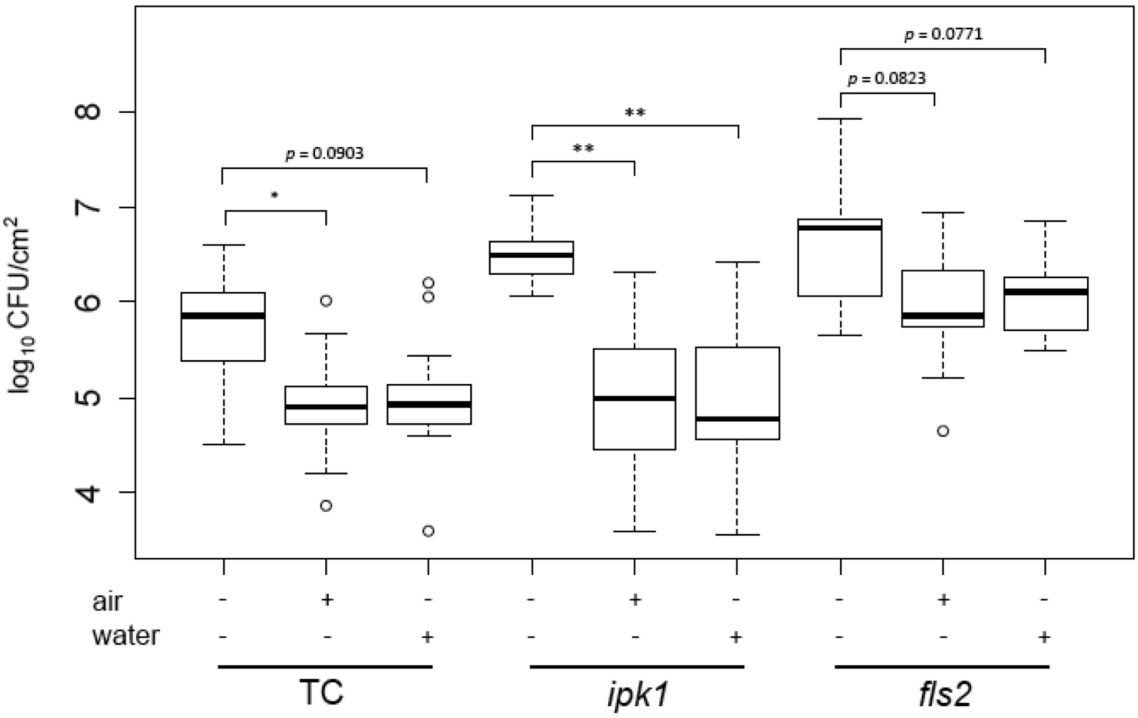

Fig. S9. Infiltration of leaves with air or water one day before challenge with *Pseudomonas syringae* induced resistance in non-mutant plants and *ipk1* mutants but not in *fls2* mutant plants. Arabidopsis leaves were infiltrated with air using an empty syringe, or infiltrated with water, or left undisturbed on day '0'. One day later, the same leaves were challenge inoculated with virulent Pst (10^5 CFU/ml). Leaf discs were sampled three days post inoculation and leaf extracts were used for bacterial serial dilution assays. Results were pooled from three experiments for statistical analysis (two leaves per plant, n = 13 - 15 plants). Asterisks denote statistically significant differences between the indicated samples (unequal variances with one-way Welch's and Games-Howell post hoc test, ** $p < 0.001$ and * $p < 0.05$).
