# Supplementary material for: Inositol hexakisphosphate biosynthesis underpins PAMP‐triggered immunity to Pseudomonas syringae pv. tomato in Arabidopsis thaliana but is dispensable for establishment of systemic acquired resistance
Source: Mol Plant Pathol. 2019 Dec 26;21(3):376–87. doi: 10.1111/mpp.12902 (PMC7036367; doi:10.1111/mpp.12902)
Supplement: Supplementary file 10 — TABLE S1 List of primers used in this study [file MPP-21-376-s010.docx]

**Table S1.** List of primers used in this study. The following abbreviations are used: qPCR, quantitative polymerase chain reaction; F, forward primer; R, reverse primer; LP, left border primer of gene of interest; and RP, right border primer.

| **Target** | **Primer sequence (5'- 3')** | **Annealing temperature (°C)** | **Application** | **Reference or Source** |
| --- | --- | --- | --- | --- |
| *CAD5* At4g34230 | F - GGGAGTTATCAACAATCCATTACA  R – TGAAGCTCCCCGTTATCACT | 55 | qPCR | Bethke *et al.* (2012) |
| *EF1a* At5g60390 | F - CGGTGCCAGTGGGACGTGTT  R – TGGCGGCACCCTTAGCTGGA | 55 | qPCR | Westwood *et al.* (2013) |
| *FRK1* At2g19190 | F - TGACGTTGGCTCGGCTTGGA  R – TGCTCGAGGAACCATCTCGGTG | 55 | qPCR | Westwood *et al.* (2013) |
| *GAPDH* At1g13440 | F - TTGCACCACTAACTGCCTTG  R - TGAGACATCAACGGTTGGA | 57 | qPCR | Westwood *et al.* (2013) |
| *IPS1* At4g39800 | F - CGTTGGGATGAACGATACAAT  R - ACATTGTTCCTGATCGCCATA | 56 | qPCR | Luo *et al.* (2011) |
| *IPS1* SALK_023626 | LP - TTGCTAGCAACCATATCGTCC  RP - TTCGTGTCGGATCTTTTAACG | 57 | T-DNA screening | SALK |
| *IPS2* At2g22240 | F - GATCATGGAGTACAAGTGAAGG  R - TAAACCGACCAGATCCATATC | 56 | qPCR | Luo *et al.* (2011) |
| *IPS2* SALK_031685 | LP - ATGGATCAACACTAACCGCTG  RP - CTCACAATCGAAGTAGGCTGC | 57 | T-DNA screening | SALK |
| *IPS3* At5g10170 | F - ACTTGTCAGCACCTCAAACA  R - ATTGCTCTCTTGCTGTCTCC | 56 | qPCR | Genscript |
| *IPS3* SALK_097807 | LP - TCACAATCGTGTTCTTCCCTC  RP - TGATCAAAGGCACCAAGAAAC | 57 | T-DNA screening | Dr. R. Le Fevre (this study) |
| *IPK1* At5g42810 | F - CAAGGAATGCTTGGGATTCAGAA  R - GATTTGTTCTGCCGTGTTTTCG | 55 | qPCR | Genscript |
| *MPK3* At3g45640 | F - AGTTGCTTGGCACACCGACAGA  R - AAGGGCTGACGTGGGAAGTTGG | 55 | qPCR | Westwood *et al.* (2013) |
| *MPK11* At1g01560 | F - GCTTCTGGCATCGTCTGTG  R - CTCAAAGTTCTCTTAGCGTCGAT | 55 | qPCR | Bethke *et al.* (2012) |
